# Distribution of axial length, anterior chamber depth, and corneal curvature in an aged population in South China

**DOI:** 10.1186/s12886-016-0221-5

**Published:** 2016-05-01

**Authors:** Hui Chen, Haotian Lin, Zhuoling Lin, Jingjing Chen, Weirong Chen

**Affiliations:** State Key Laboratory of Ophthalmology, Zhongshan Ophthalmic Center, Sun Yat-sen University, Guangzhou, Guangdong 510060 China

**Keywords:** Axial length, Anterior chamber depth, Corneal curvature

## Abstract

**Background:**

Ocular biometry is important for preoperative assessment in cataract and anterior segment surgery. The purpose of this study was to investigate normative ocular biometric parameters and their associations in an older Chinese population.

**Methods:**

This was a cross-sectional observational study. From 2013 to 2014, we recruited inhabitants aged 50 years or older in Guangzhou, China. Among 1,117 participants in the study, data from 1,015 phakic right eyes were used for analyses. Ocular parameters including axial length (AL), anterior chamber depth (ACD), and corneal curvature (K) were measured using an IOL Master.

**Results:**

The mean AL, ACD, and K were 23.48 mm [95 % confidence interval (CI), 23.40–23.55], 3.03 mm (CI, 3.01–3.05), and 44.20 mm (CI, 44.11–44.29), respectively. A mean reduction in ACD with age was observed (*P* = 0.002) in male subjects but not in female subjects (*P* = 0.558). Male subjects had significantly longer ALs (23.68 mm versus 23.23 mm, *P* < 0.001), deeper ACDs (3.13 mm versus 2.95 mm, *P* < 0.001), and flatter Ks (43.85D versus 44.50 D, *P* < 0.001) than female subjects. Eyes with axial elongation had a flatter cornea (*r* = −0.437, *P* < 0.001) and a deeper anterior chamber (*r* = 0.652, *p* < 0.001). The ACD was correlated with K (*r* = −0.266, *P* < 0.001).

**Conclusions:**

These data provide normative values for AL, ACD, and K using the IOL Master for a population in South China. The AL in this Chinese cohort was greater than that observed in the Singaporean Chinese but smaller than that observed in Malaysia and for Caucasians. The Chinese have a shallower ACD than some other racial groups. Age and sex were the most consistent predictors of ocular biometry in the older population from South China.

## Background

Axial length (AL) is one of the basic anatomical parameters in ophthalmology, and a major variable for refractive error and for diagnosing certain pathological conditions such as staphyloma and the risk of retinal detachment before refractive surgery [1–5]. Cataract surgery has become one of the most commonly performed surgical procedures worldwide, and AL and other ocular biometrics such as anterior chamber depth (ACD) and corneal curvature (K) are considered the most important determinants for accurate intraocular lens (IOL) power calculations. Determination of the normal range of these parameters will provide ophthalmologists with important information that can lead to an improvement in cataract surgical outcomes.

In our recent study of the adult Chinese population before cataract surgery, we reported that the standard deviation of the AL in this cohort was lower than European populations. Furthermore, there was no statistically significant difference in AL between age groups [6]. However, the study was clinic based, and may not be representative of the entire population.

To avoid the potential selection bias present in specific and highly selective patient groups, we now describe the distribution of AL and its associations with ocular biometric parameters measured with the IOL Master in a community-based survey. These data will provide comprehensive age and sex normative data for this instrument in the older South China population.

## Methods

### Study population

In this population-based, cross-sectional study, subjects older than 50 years of age who did not have any serious ophthalmic disorders were recruited from four community free clinics. The free clinics were in parks from the Yuexiu and Tianhe Districts of Guangzhou. They provide primary eye care services for inhabitants who participate in daily outdoor activities in the park. Subjects were approached at random by healthcare workers and invited for a primary eye care services at the free clinics. These inhabitants were selected because they were a stable and an older population that was representative of Guangzhou. The subjects were identified by systematic sampling (every fifth patient registered at the free clinic) and asked to participate in the study after they provided written informed consent. A detailed questionnaire including demographic information, socioeconomic details, and medical and ocular histories was administered.

Examination of subjects was performed between September 2013 and July 2014. All study procedures were performed in accordance with the tenets of the Declaration of Helsinki, and the study was approved by the Ethics Committee of the Zhongshan Ophthalmic Center.

### Procedures

**Fig. 1:**
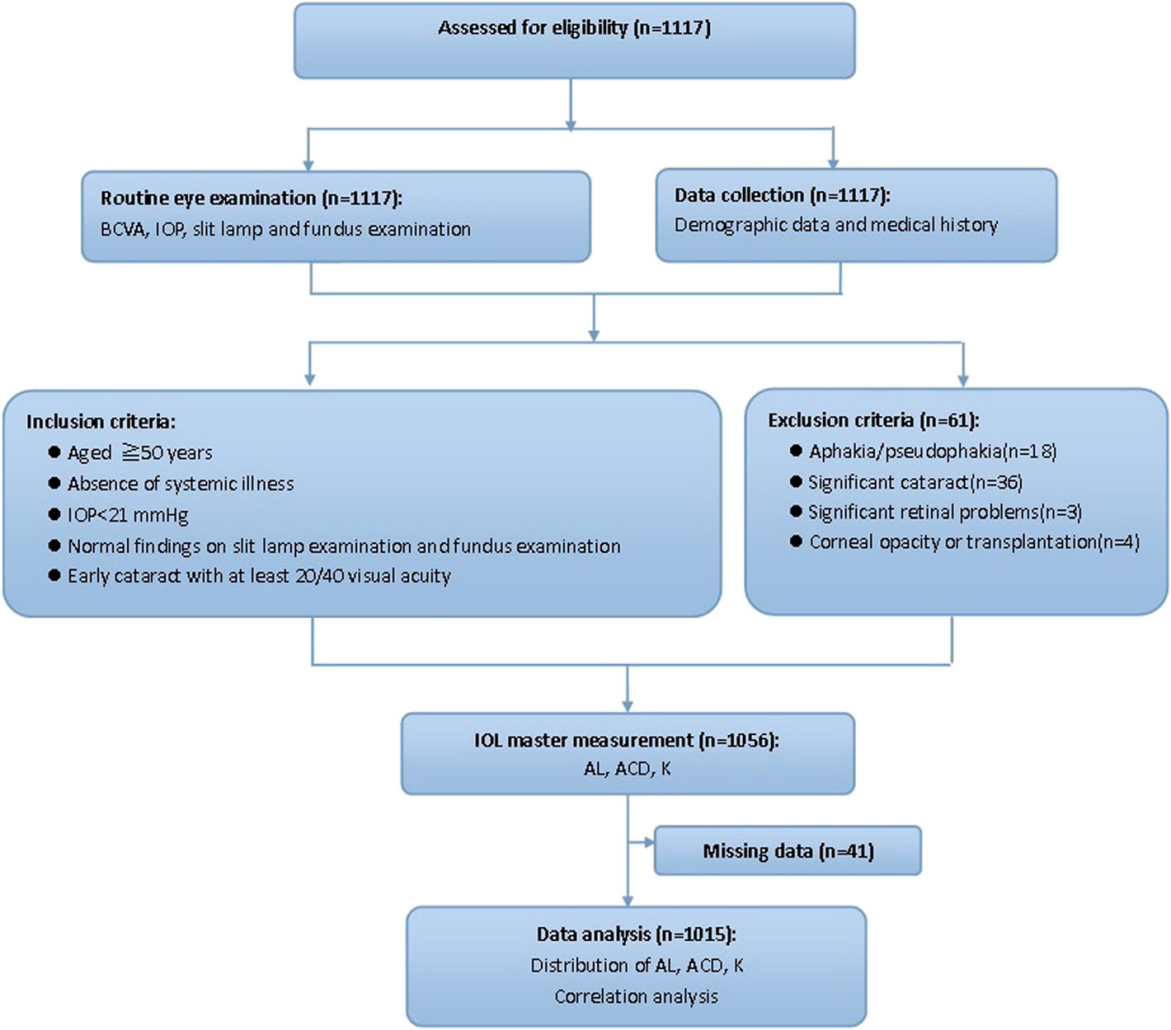
Summary of the trial design

A summary of the enrollment and study procedures is shown in Fig. 1. All participants had a standardized slit lamp examination. Inclusion criteria were as follows: the absence of uncontroled systemic illness, intraocular pressure of 21 mmHg or less, and normal findings on slit lamp examination and fundus examinations. In eyes with early cataracts, only patients with at least 20/40 visual acuity were included. Exclusion criteria were previous ocular surgery, a history of corneal disease, contact lens wear, and dry eye that complicated the examination. The examinations were performed by skilled operators who were trained before the study.

The AL, ACD, and K were measured with noncontact partial coherence laser interferometry (IOL Master, version 3.01; Carl Zeiss Meditec, Jena, Germany). The validity and repeatability of the IOL Master measurements have been previously reported [1]. AL measurements were performed a minimum of five times in each eye (minimum signal/noise ratio of 100), and a standard deviation of 0.15 mm for AL was required. Three keratometric measurements were made in an automated mode and the mean calculated by the device was used for further analyses. Corneal power was measured in two meridians, the greatest and least radii of curvature (K1, K2). Keratometric data were reported as corneal power in diopters (D). The refractive index value used for this conversion by the IOL Master was *n* = 1.3375. Subsequent to keratometric measurements, ACD was also measured a minimum of three times, and 0.13 mm for ACD was required for optimal scans. If subjects had poor visual acuity, poor fixation and high myopia which would make measurements varied, then measurements were repeated until reproducible results were obtained. All patients were tested by the same experienced examiner.

### Statistical analysis

Statistical analysis was performed using SPSS software, version 19.0 (SPSS, Chicago, IL, USA). The Kolmogorov–Smirnov test was used to evaluate the normality of the distribution for all variables. Because the biometric data of the left and right eyes were similar (*P* > 0.05), the right eyes were arbitrarily chosen to represent a specific individual. Analysis of variance was conducted to evaluate the variation in different biometric components. Correlations between AL and other biometric variables were calculated using Pearson’s correlation coefficient, and 95 % confidence intervals (CIs) were reported. Group trend tests were used to assess any significant trends across age groups for each variable.

## Results

We initially examined 1,117 subjects. Data from 102 subjects who did not fulfill the study criteria were eliminated, including aphakia/pseudophakia (18 eyes), significant cataracts (36 eyes), significant retinal problems (three eyes), corneal opacity or transplantation (four eyes), and 41 additional subjects who had missing data. Finally, data from 1,015 subjects were analyzed. There were no differences between subjects included in our analyses and those excluded because of age (*P* = 0.064) or sex (*P* = 0.254).

**Fig. 2:**
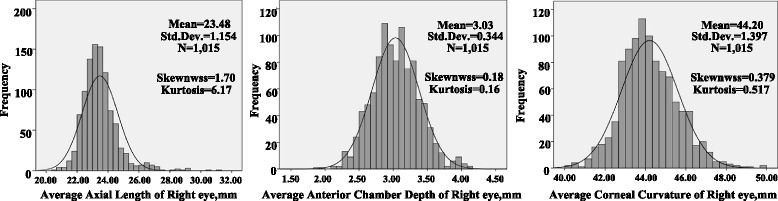
Distribution of axial length, anterior chamber depth, and corneal curvatures

The mean AL was 23.48 mm (95 % CI, 23.40–23.55), the mean ACD was 3.03 mm (95 % CI, 3.01–3.05), the mean K1 was 43.79 D (95 % CI, 43.70–43.87), the mean K2 was 44.63 D (95 % CI, 44.54–44.72), and the mean K was 44.20 D (95 % CI, 44.11–44.29). Table 1 shows the mean AL and other biometric parameters stratified by age and sex. Figure 2 shows a histogram of the distribution of AL, ACD, and K. The Kolmogorov–Smirnov test results indicated a significant difference from normal distributions (*P* < 0.05) of AL and K. According to the distribution indices, AL and K had leptokurtic distributions.

**Table 1:** Age and gender distribution of ocular biometry measures with 95 % confidence intervals

				CC(D)			
Age group(years)	*n*	AL(mm)	ACD(mm)	K1	K2	K	ΔK
Gender							
Men							
50–59	80	23.61(20.93–28.84)	3.14(2.27–4.01)	43.75(40.08–46.68)	44.43(41.11–48.28)	44.08(40.59–47.47)	0.68(0.06–3.05)
60–69	244	23.83(20.89–30.34)	3.15(2.27–4.01)	43.41(36.49–49.06)	44.21(41.16–50.45)	43.81(40.23–49.74)	0.80(0.00–8.33)
70–79	117	23.97(21.40–29.03)	3.13(1.97–4.16)	43.26(39.57–47.07)	44.25(40.56–48.35)	43.75(40.30–47.70)	0.99(0.11–3.57)
80+	26	23.54(22.03–24.92)	2.91(1.90–3.59)	43.23(39.99–46.30)	44.86(42.94–47.8)	44.02(41.46–46.97)	1.63(0.29–3.14)
All	467	23.81(20.89–30.34)	3.13(1.90–4.16)	43.42(36.49–49.06)	44.29(40.56–50.45)	43.85(40.23–49.74)	0.87(0.00–8.33)
Women							
50–59	117	23.04(20.88–31.15)	2.93(2.14–4.10)	44.16(40.42–48.49)	44.92(42.45–49.71)	44.54(42.06–49.09)	0.76(0.00–4.64)
60–69	289	23.22(20.62–29.27)	2.96(2.14–4.10)	44.11(40.76–47.94)	44.89(41.31–49.41)	44.49(41.03–48.35)	0.78(0.00–3.42)
70–79	129	23.26(21.53–29.23)	2.96(2.19–3.95)	43.98(40.27–48.01)	44.92(40.52–49.56)	44.44(40.39–48.77)	0.94(0.16–3.81)
80+	13	23.13(22.27–24.54)	2.88(2.28–3.62)	44.25(41.36–49.13)	45.18(42.24–50.22)	44.71(41.80–49.67)	0.93(0.24–2.38)
All	548	23.19(20.62–31.15)	2.95(2.14–4.10)	44.09(40.27–49.13)	44.91(40.52–50.22)	44.50(40.39–49.67)	0.82(0.00–4.64)
Whole population							
50–59	197	23.27(20.88–31.15)	3.02(2.14–4.10)	43.99(40.08–48.49)	44.72(41.11–49.71)	44.35(40.59–49.09)	0.73(0–4.64)
60–69	533	23.50(20.62–30.34)	3.04(2.10–4.01)	43.64(36.49–49.06)	44.58(41.16–50.45)	44.18(40.23–49.74)	0.79(0–8.33)
70–79	246	23.60(21.40–29.23)	3.04(1.97–4.16)	43.57(39.57–48.01)	44.60(40.52–49.56)	44.11(40.30–48.77)	0.96(0.11–3.81)
80+	39	23.41(22.03–24.92)	2.90(1.90–3.62)	43.78(39.99–49.13)	44.96(42.24–50.22)	44.25(41.46–49.67)	1.40(0.24–3.14)
All	1015	23.48(23.40–23.55)	3.03(3.01–3.05)	43.79(43.70–43.87)	44.63(44.54–44.72)	44.20(44.11–44.29)	0.84(0.80–0.88)

*ACD* anterior chamber depth, *AL* axial length, *CC* corneal curvature, *D* diopters, *mm* millimeters

**Fig. 3:**
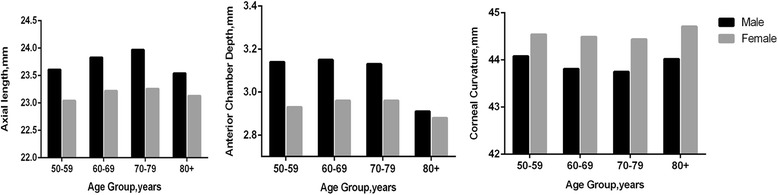
Frequency distribution of the sample by age group

The ACD decreased across age groups in male subjects (*P* = 0.002), but there was no significant age-related change in AL and K (*P* = 0.411, *P* = 0.614, respectively) (Fig. 3). After adjustment for age, the AL was significantly longer in male subjects than in female subjects (23.81 mm versus 23.19 mm; *P* = 0.020). The mean ACD was deeper in male (3.13 mm) than in female subjects (2.95 mm; *P* < 0.001). Female subjects had a steeper K than male subjects (44.50 mm versus 43.85 mm; *P* < 0.001).

ACD correlated positively with AL (*r* = 0.652, *P* < 0.001). The K was correlated with ACD (*r* = −0.266, *P* < 0.001). There was also a significant negative correlation between AL and K (*r* = −0.437, *P* < 0.001) and a higher astigmatism was associated with shallower ACD (*r* = −0.064, *P* < 0.05).

## Discussion

Our study provides population-based cross-sectional normative data on AL, ACD, and K based on IOL Master measurements of an adult population, 50–98 years of age, from urban South China. In this study, we confirmed previous data that AL and ACD were not normally distributed in the older population [7]. We also found that older male subjects had a shallower ACD, and female subjects had a shorter AL, shallower ACD, and steeper K than male subjects. These patterns are similar to those observed in a Chinese population study conducted in Singapore and mainland China that used similar protocols but measured biometry with A-scan ultrasound, rather than the IOL Master [8, 9]. However, as we described in a previous study, we did not observe any age-specific differences in AL and K in this population [6].

The normal ranges of ocular parameters are important in the formulas used for intraocular lens calculations for cataract surgery. However, the distribution of AL in the normal population in South China determined by the IOL Master has not been reported. The mean AL in the present study (23.48 mm) was shorter than that of cataractous eyes (23.60 mm) reported in our previous study. When compared with other population-based surveys (Table 2), it is longer than reported in the Tanjong Pagar Study in Singaporean Chinese (23.23 mm), in the Liwan Study in South China (23.11 mm) [8, 9], and also longer than the studies in India [10], Mongolia [11], and Burma [12]. It was almost identical to the AL of Caucasians [7, 13], but shorter than the AL reported in the Beaver Dam Eye Study [(BDES); 23.69 mm] and in the EPIC-Norfolk Study from England (23.80 mm) [14, 15]. These differences may be due to different calibrations of the devices such as the IOL Master and ultrasound used to obtain biometry values. In addition, our study showed that the AL distribution was slightly skewed to the right and had a leptokurtic distribution. This finding is consistent with data from previous studies of refractive error in China [3, 9]. The abnormal distribution of greater AL could be attributed to the high myopic refractive error in this population.

**Table 2:** Mean axial length, anterior chamber depth, corneal and curvature radius reported in population-based studies

			AL			ACD			CC		
Study	Ethnicity	Measurement	Men	Women	All	Men	Women	All	Men	Women	All
The Los Angeles Latino Eye Study	Latinos	Ultrasound	23.65	23.18	23.38	3.48	3.36	3.41	43.35	43.95	43.72
The Mongolian Study	Mongolians	Ultrasound	23.43	23.08	23.13	2.87	2.77		43.65	44.24	
The Beaver Dam Eye Study	white	IOL Master	23.92	23.51	23.69	3.14	3.09	3.11	7.77^a^	7.65^a^	7.70^a^
The Meiktila Eye Study	Burmese	Ultrasound	23.12	22.54	22.76	2.86	2.79	2.82	7.71^a^	7.56^a^	7.62^a^
The Reykjavik Study	White	Ultrasound	23.74	23.20		3.20	3.08		43.41	43.73	
The Liwan Eye Study	Chinese	Ultrasound	23.38	22.83	23.11	2.75	2.61	2.67	43.50	44.25	43.88
The tanjong pagar study	Chinese	Ultrasound	23.54	22.98	23.23	2.99	2.81	2.90	7.73^a^	7.59^a^	7.65^a^
The Singapore Malay Eye Study	Malay	IOL Master			23.55			3.10			7.65^a^
The Singapore Indian Eye Study	Indians	IOL Master	23.68	23.23	23.45	3.19	3.10	3.15	7.68^a^	7.55^a^	7.61^a^
The blue mountain eye study	white	IOL Master	23.75	23.20	23.44	3.16	3.06	3.10	43.01	43.74	43.42

*ACD* anterior chamber depth, *AL* axial length, *CC* corneal curvature;

^a^Corneal curvature in mm

The mean ACD (3.03 mm) from our study is deeper than in the Chinese, Liwan, (2.67 mm), and Tanjong Pargar Studies (2.90 mm), but shallower compared with the Caucasian population (3.11 mm) [7–9]. Compared with the A-scan ultrasound, AL measured by the IOL Master has been reported to be significantly longer and more accurate [16–18]. ACD measured by ultrasound was found to be significantly shorter compared with noncontact measuring systems [19]. Additionally our population was slightly older.

In contrast to the trend of longer ALs in younger people from other populations, we did not observe any age-specific differences in ALs in our population [8, 11, 20]. Consistent with our study, the Los Angeles Latino Eye Study and the Liwan Study did not show an association between AL and age [9, 13]. However, the AL increased with increasing age in the Central Indian Eye and Medical Study and the Mongolian Study [10, 11]. One explanation for this discrepancy is that the age-related differences in AL may be attributed to a cohort effect rather to an actual reduction of AL with age. It is possible that once the eye has achieved its adult size, insignificant change occurs in the AL during adulthood aging. In the BDES and The Singapore Malay Eye Study (SiMES), after adjusting for height and education, the association between age and AL was insignificant, suggesting a cohort effect caused by socioeconomic background and stature development [14, 21]. ACD was also negatively correlated with age in male but not in female subjects, whereas ACD has been reported to decrease with age in both sexes in other populations [7, 21]. The age-specific trend in ACD can be explained by an age-related increase in lens thickness [22]. It is also possible that the flattening of the ACD observed in our studies may result from a cohort effect involving younger individuals, especially male subjects, who may be exposed to more near work and become more myopic with a deeper ACD.

In our study, there was no correlation between K and age. However, a trend for age-related increases of K has been reported in young subjects. Hayashi et al. reported that the degree of both horizontal and vertical corneal curvatures increases with age [23]. Consistent with our study, Lee et al. reported that after adjusting for the effects of other parameters, changes in corneal curvature did not have a statistically significant correlation with age [24].

Regarding sex variations, we observed that male subjects had longer eyes, deeper anterior chambers, and flatter corneas than female subjects as measured by the IOL Master. The BDES reported that male subjects had longer ALs and larger eyes, but these differences were not significant after adjustment for height [14]. However, the SiMES reported that sex differences in AL and ACD were still significant when using multivariate analyses and controlling for stature, suggesting that sex may be an independent determinant of the AL [21]. Taken together, the results from multiple reports suggest that genetic and other factors may account for the differences in biometry between male and female subjects.

In our study, the AL and ACD were significantly correlated, indicating that a longer AL or higher levels of myopia are associated with steeper corneas. We also found a significant negative correlation of K with AL, which has been reported in other studies [25, 26]. The inverse correlation with AL can be attributed to the emmetropization mechanism that compensates for a higher AL by decreasing the corneal power [27]. In addition, higher astigmatism was associated with steeper ACD. To our knowledge, this relationship has not been previously reported.

Our study provides population-based data and examines a homogeneous population. It avoids a potential bias associated with clinic-based studies, and excludes confounding variables such as small sample size and differing methodologies [28]. Furthermore, the biometric measurements were performed by the IOL Master that has been proven to be a better predictor of normative ocular biometric data than ultrasound biometry [29]. However, the cross-sectional nature of our study is a limitation in assessing age-related changes of biometric characteristics because any apparent trend may result from a cohort effect rather than a longitudinal age-related change.

## Conclusions

In conclusion, this study provides normative ocular biometry data and their interrelationships for a large representative older population in South China. Age and sex variations in ocular biometry were observed in this population, with older people generally having a shallower ACD and flatter K, while male subjects had longer eyes, deeper anterior chambers, and flatter corneas than female subjects. Eyes with axial elongation tended to have flatter corneas and deeper anterior chambers. Furthermore, measurements by the IOL Master indicated that age and sex were the most consistent predictors of ocular biometry in the adult population of South China.

### Ethics approval and consent to participate

All study procedures were performed in accordance with the tenets of the Declaration of Helsinki, and the study was approved by the Ethics Committee of the Zhongshan Ophthalmic Center. Written informed consent was obtained from all the study participants.

### Consent for publication

Not applicable.

### Availability of data and materials

All the data supporting the conclusions of this article is included within the article.

## Abbreviations

ACD: anterior chamber depth
AL: axial length
BDES: Beaver Dam Eye Study
D: diopters
IOL: intraocular lens
K: corneal curvature
SiMES: The Singapore Malay Eye Study
